# Advancing nursing students' transition to scholarship: embedding a librarian into the Advanced Nursing Research course

**DOI:** 10.5195/jmla.2022.1227

**Published:** 2022-04-01

**Authors:** Viktoriya Pleshkan, Irma Singarella

**Affiliations:** 1 vyplshkn@memphis.edu, Assistant Professor, Loewenberg College of Nursing, University of Memphis, Memphis, TN; 2 irma.singarella@memphis.edu, Associate Professor, Health Sciences Librarian, University of Memphis, Memphis, TN

**Keywords:** embedded librarian, nursing education, survey, library services, awareness, online education

## Abstract

**Background:**

Nursing students often prioritize learning clinical skills rather than research skills, possibly inhibiting their growth as scholars. Supporting nursing students' learning of information literacy skills has been shown to impact nurses' involvement with research after graduation. This suggests a need for developing innovative information literacy teaching strategies that can enable nursing students to better understand the process of research and how to apply research to practice.

**Case Presentation:**

This article describes the implementation of the embedded librarian project at the course level at the University of Memphis. A librarian was integrated into the Advanced Nursing Research course, a semester-long course for graduate nursing students, for the fall 2020 semester. This case shares the embedded librarian project's implementation and evaluation strategies.

**Conclusions:**

The embedded librarian project aided students' acquisition of information literacy skills at the University of Memphis. Students reported that the embedded librarian project helped them complete assignments for their research course. Using an embedded librarian service within the graduate nursing curricula model may enhance scholarship among future nurses.

## BACKGROUND

Nurses are highly skilled, licensed health care professionals who play an important role in patients' disease prevention and health promotion. Professional practice guidelines note that nurses' clinical practice should be evidence based, yet nursing students' stated interest and intention on using research as clinicians is often low. This finding is particularly important given that evidence suggests that a nursing student's intention on using evidence correlates with their eventual use of research in their clinical practice after graduation [1]. Because both interpreting and generating research require sufficient information literacy skills, nursing educators should consider exploring novel approaches that may improve nursing students' information literacy skills.

In 2013, the Association of Colleges and Research Libraries (ACRL) established the Information Literacy (IL) Competency Standards for Nursing [2, 3]. The IL Standards for Nursing outline the skills needed to engage in evidence-based nursing. The IL Standards for Nursing also provide a common language that nursing faculty and librarians can use when discussing student information skills, which can be used to foster the collaborative development of learning activities that will support students' learning of information-seeking skills. The ACRL also provides guidelines for the provision of library services to distance learners, which state that online students must receive library services equivalent to those provided for students on campus [2].

The University of Memphis is an urban, public research university with a Carnegie classification of Doctoral Universities: High Research Activity. The University of Memphis includes the Loewenberg College of Nursing, which offers both baccalaureate and master's programs that are accredited by the Commission of Collegiate Nursing Education (CCNE) and approved by the Tennessee Board of Nursing. At the University of Memphis Libraries, each librarian is assigned as liaison to one or more academic department(s). In this role, librarians are expected to become familiar with the teaching and research needs of the department(s) to inform collection development and research services.

Librarians interested in collaborating with faculty through online courses often participate as embedded research consultants for both graduate and undergraduate students. They are often tasked with creating course-specific materials and providing research consultations to students and faculty [4]. Embedded librarians can facilitate virtual students' learning experience by increasing students' comfort with their research assignments and by being available at the point of need [5, 6].

Because all care must be evidence based, nurses must be knowledgeable of how to use and generate knowledge, which leads many nursing programs to develop courses aimed at promoting nursing students' research skills. Offering library consultations to students enrolled in research courses may decrease students' anxiety and improve students' confidence when working on their research assignments [7]. Courses focused on research may prove especially challenging to nursing students returning to school for an advanced nursing degree, as these students may have very limited experience with how to translate the skills necessary to complete an academic research assignment into a clinical workplace environment [7]. Providing these students with the additional support of an embedded librarian may lead to their increased involvement with research as clinicians, as nurses' involvement with research can lead to increased evidence-based practice and practice-based research [1].

## CASE PRESENTATION

The embedded librarian project at the University of Memphis was implemented in the fall of 2020 to assist graduate nursing students in their semester-long Advanced Nursing Research course. The Advanced Nursing Research course objectives included locating current and relevant evidence for nursing research questions, as well as critiquing published research studies as sources of evidence for clinical decision-making [8]. Major course assignments consisted of a population, intervention, comparison, outcome, timing (PICOT) research question, quantitative and qualitative research critique papers, and a literature review paper [8].

In previous iterations of this course, students had struggled with searching the literature and distinguishing between quantitative and qualitative research. We sought to assess whether an embedded librarian could help students develop more refined information retrieval and critical assessment skills. A nursing librarian, who was embedded into the course via the University of Memphis D2L learning management system and had access to students' assignments, understood the students' specific learning needs pertaining to the course. Services available to faculty and students in this course included helping locate and evaluate relevant research studies, creating and linking to course-specific library resources, and communicating any issues or additional students' needs during the semester.

In addition to having access to the university libraries, all twenty Advanced Nursing Research course students had access to a course-specific research guide, which was integrated into the course page in the learning management system. The course research guide was customized to meet the students' course-specific needs through the collaborative effort of the course professor and the librarian. Some of the topics in the guide included information on quantitative and qualitative research methods, literature search strategies, APA writing style, and research question structure [9, 10]. The students were encouraged to review the guide to help them with their written paper assignments. During the fall of 2020, this course research guide was accessed 166 times. The research guide was accessed most during the months of September and October, which coincided with the students' paper assignments (PICOT, quantitative and qualitative papers).

In addition to the research guide, students had several opportunities to connect with their embedded librarian, who assisted them in locating and evaluating relevant research information. The nursing librarian assisted students in searching and evaluating their research-relevant studies based on the assignments' criteria. She supported the students in the process of accessing the literature, identifying the proper keywords, and recovering the relevant resources. The librarian demonstrated to students the diverse search strategies and possible keyword combinations. She oriented the students on the use of database features such as filters, citation tools, and how to find similar articles. To assist students with their three paper assignments, the embedded librarian presented students with different examples of both qualitative and quantitative methodologies. This allowed students to gain further understanding of their research topics. Additionally, the embedded librarian used both group and individual Zoom meetings for research consultations to accommodate students' schedules. Ten students attended group meetings, but many other students preferred individual research consultations. During the fall semester of 2020, the librarian conducted fifty-one individual sessions for students, providing them with research advice and training them in the use of library databases.

### Program evaluation

As part of the course program evaluation, an anonymous end-of-course survey (supplementary materials) was developed by the embedded librarian and the course faculty. The survey was distributed through the Desire2Learn learning management system to each of the students enrolled in the course (n=20). The students were asked to answer eight questions, including six 5-point Likert scale questions (to rate their agreement with a statement) and two open-ended questions about their use of and satisfaction with the embedded librarian services. This survey protocol was reviewed by the University of Memphis Institutional Review Board (IRB), which determined that this project did not meet the Office of Human Subjects Research Protections' definition of human subjects research and 45 CFR part 46 does not apply. As such, this study was deemed not subject to IRB review.

Fourteen students voluntarily responded to the survey, a response rate of 70%. Most students indicated that working with the course librarian helped them to become better at researching health sciences literature, helped complete course assignments, improved their grades, led to an improvement in the quality of their projects, and helped them to feel more confident about their research abilities (Table 1).

**Table 1:** Embedded librarian project Likert survey results

Survey question	Students' answers
Working with an embedded librarian has …	agree to strongly agree
helped me become better at searching health sciences literature	85.7% (n=12)
assisted me in class	78.6% (n=11)
helped me complete assignments	78.6% (n=11)
led to improvement in my grades	71.4% (n=10)
led to improvement in the quality of my projects	78.6% (n=11)
helped me feel more confident about my research abilities	78.6% (n=11)

Additionally, students were asked to note three things they liked about the embedded librarian project and three things they would like to see improved (Table 2). Descriptive analysis of the 5-point Likert scale data focused on the answers' frequencies and percentages extracted from the D2L survey statistical data. We analyzed students' responses to the open-ended questions using a summative content analysis method. We compressed these open text responses into fewer categories by using summative content analysis [11], which can be used to interpret the meaning of the textual qualitative data [12]. Students' answers were analyzed for words, pattern frequencies, and meaning [12]. Categories with similar meanings were identified to describe students' experiences with the embedded librarian project. Gaining the information about students' experiences through these open-ended questions allowed us to collect direct data from participants without setting preconceived categories [12]. At the end of the course as part of the data analysis, students expressed appreciation of these library resources and the librarian's flexible schedule.

**Table 2:** Embedded librarian project open-ended questions survey results

Survey question	Students' answers
Please list 3 things that you liked about the embedded librarian option	Availability, accessibility, flexibility, quick feedback, librarian expertise
Please list 3 things that you would like to see improve or change regarding the embedded librarian option	Increase availability, improve online access to the librarian

## DISCUSSION

Research training can have a substantial impact on nurses' use of research in practice after graduation, but others have found that nurses are often reluctant to explore their own research questions, instead preferring to focus on more practical nursing applications [13, 1]. In our Advanced Nursing Research course, most students drew their research ideas from their current practice, utilizing very little research literature to decide on their paper research question. Moreover, through our interactions with the students, we anecdotally noticed the lack of motivation to do research at the beginning of the course among students enrolled in our research course. Students' motivation is strongly connected to their feeling of competence and meaning, which can be fostered through teacher-student interactions [14]. Though students did not seem to see the merit of completing their paper assignments at the beginning of the course, they seemed to be much more motivated after they submitted their research question (PICOT) paper. Because each student based their research question on their individual clinical interests, it is assumed that this research paper experience became more meaningful to students. Some students emailed their teacher commenting that they are making a difference in their practice areas in real time by completing their research papers. Though the course assignments appeared to be meaningful to students, students' sense of competence was harder to develop. We believe that the embedded librarian project assisted students in developing information literacy skills, which may have an impact on their perceived competency.

Our project data showed that many students indicated that they specifically appreciated their librarian's availability, accessibility, and knowledge of health sciences. Students also suggested including late office hours to increase the librarian's availability, which aligns with previous studies that found that online students would like to have access to a librarian at any time [15]. Our nursing students found that having a librarian who understood their assignments and who was readily available within their “digital backyard” was very helpful in completing their research course assignments.

To lower barriers to participating in research, nursing students need additional training on how to find, read, and utilize existing evidence [13]. The most valuable component of the embedded librarian project reported by our students was the librarian's help with searching the literature, which aligns with findings reported elsewhere in the literature [6]. More than 85% of students in our survey reported that this embedded librarian project assisted them in becoming better at searching health sciences literature. While the evidence demonstrating the impact of embedded librarians remains limited, case presentations such as this can help address this by highlighting how nursing students in a research course may benefit from additional opportunities to work closely with a librarian [4].

Nursing students' use of research when transitioning to practice does not happen automatically; rather, the ability to contribute to the science of nursing must be supported and developed. The diversity and accessibility of the embedded librarian online resources encouraged students' active involvement in this graduate research course. While this approach did not address students' motivational and attitudinal barriers to research, students' overall feedback suggested that the embedded librarian service was valuable. Projects such as this may increase the likelihood of future nurses' involvement with research [1].

This project draws from a small convenience sample gathered at a single institution, which limits the generalizability of its findings. However, because limited information is available in the literature on the impact of embedded librarianship [4], this project's results contribute to the understanding of the importance of utilizing an embedded librarian in nursing programs. Future studies should focus on nursing students' perception of scholarship and directly assessing students' research competencies, as well as correlating students' performance in their research courses with their utilization of the embedded librarian.

## Data Availability

The data generated and/or analyzed during the current study are not publicly available for legal/ethical reasons but are available from the corresponding author on reasonable request.

## References

[1] Forsman H, Wallin L, Gustavsson P, Rudman A. Nursing students' intentions to use research as a predictor of use one year post graduation: a prospective study. Int J Nurs Stud. 2012 Sep 1;49(9):1155–64. DOI: 10.1016/j.ijnurstu.2012.04.002. 22564505

[2] Association of College & Research Libraries (ACRL). Standards for distance learning library services [Internet]. The Association; 2006 [cited Oct 2021]. <www.ala.org/acrl/standards/guidelinesdistancelearning>.

[3] Association of College & Research Libraries (ACRL). Information literacy competency standards for nursing [Internet]. The Association; 2013 [cited Oct 2021]. <www.ala.org/acrl/standards/nursing>.

[4] Schulte SJ. Embedded academic librarianship: a review of the literature. Evid Based Libr Inf Pract. 2012 Dec;122–38. Available at: http://ejournals.library.ualberta.ca/index.php/EBLIP/article/view/17466/14483.

[5] Hoffman S, Ramin L. Best practices for librarians embedded in online courses. Public Services Quarterly. 2010 Sep 14;6(2–3):292–305. DOI: 10.1080/15228959.2010.497743.

[6] Wu L, Betts VT, Jacob S, Nollan R, Norris T. Making meaningful connections: evaluating an embedded librarian pilot project to improve nursing scholarly writing. J Med Libr Assoc. 2013 Oct;101(4):323. DOI: 10.3163/1536-5050.101.4.016. 24163606 PMC3794690

[7] Zanin-Yost A, Dillen C. Connecting past to future needs: nursing faculty and librarian collaboration to support students' academic success. J Libr Adm. 2019 Jan 2;59(1):45–58. DOI: 10.1080/01930826.2018.1549407.

[8] Pleshkan V. Syllabus for Advanced Nursing Research. Memphis, TN: Department of Nursing, University of Memphis; fall 2021.

[9] University of Memphis Libraries Tutorial and research guide. Advanced Nursing Research [YouTube]. 2020 [cited 20 September 2021]. Available from: https://www.youtube.com/playlist?list=PLrg_dpZ2-LyxA-bC1EVrdvcREY9X2U4ez.

[10] Singarella I, McClue J. Tutorial and research guide. Advanced Nursing Research [Internet]. University of Memphis Libraries; 2020. <https://libguides.memphis.edu/NURS7002AdvancedNursingResearch/home>.

[11] Cho JY, Lee EH. Reducing confusion about grounded theory and qualitative content analysis: similarities and differences. Qual Rep. 2014 Aug 11;19(32). DOI: 10.46743/2160-3715/2014.1028.

[12] Hsieh HF, Shannon SE. Three approaches to qualitative content analysis. Qual Health Res. 2005 Nov;15(9):1277–88. DOI: 10.1177/1049732305276687. 16204405

[13] Ax S, Kincade E. Nursing students' perceptions of research: usefulness, implementation and training. J Adv Nurs. 2001 Jul 22;35(2):161–70. DOI: 10.1046/j.1365-2648.2001.01833.x. 11442695

[14] Seifert T. Understanding student motivation. Educational Research. 2004 Jun 1;46(2):137–49.

[15] Faulk N, Crist E. A mixed-methods study of library communication with online students and faculty members. Coll Res Libr. 2020 Apr 3;81(3):361. DOI: 10.5860/crl.81.3.361.

